# G-NEST: a gene neighborhood scoring tool to identify co-conserved, co-expressed genes

**DOI:** 10.1186/1471-2105-13-253

**Published:** 2012-09-28

**Authors:** Danielle G Lemay, William F Martin, Angie S Hinrichs, Monique Rijnkels, J Bruce German, Ian Korf, Katherine S Pollard

**Affiliations:** 1 Genome Center, University of California Davis, 451 Health Science Dr, Davis, CA, 95616, United States of America; 2 Department of Food Science and Technology, University of California Davis, One Shields Avenue, Davis, CA, 95616, United States of America; 3 Center for Biomolecular Science and Engineering, University of California Santa Cruz, 1156 High St, Santa Cruz, CA, 95064, United States of America; 4 Department of Pediatrics, Children’s Nutrition Research Center, Baylor College of Medicine, 1100 Bates Street, Houston, TX, 77030, United States of America; 5 Gladstone Institutes, Division of Biostatistics and Institute for Human Genetics, University of California San Francisco, 1650 Owens St, San Francisco, CA, 94158, United States of America

**Keywords:** Computational biology, Genomics, Gene expression, Gene duplication, Transcription, Cluster analysis, Gene neighborhood, Gene cluster, Bioinformatics, Evolution

## Abstract

**Background:**

In previous studies, gene neighborhoods—spatial clusters of co-expressed genes in the genome—have been defined using arbitrary rules such as requiring adjacency, a minimum number of genes, a fixed window size, or a minimum expression level. In the current study, we developed a Gene Neighborhood Scoring Tool (G-NEST) which combines genomic location, gene expression, and evolutionary sequence conservation data to score putative gene neighborhoods across all possible window sizes simultaneously.

**Results:**

Using G-NEST on atlases of mouse and human tissue expression data, we found that large neighborhoods of ten or more genes are extremely rare in mammalian genomes. When they do occur, neighborhoods are typically composed of families of related genes. Both the highest scoring and the largest neighborhoods in mammalian genomes are formed by tandem gene duplication. Mammalian gene neighborhoods contain highly and variably expressed genes. Co-localized noisy gene pairs exhibit lower evolutionary conservation of their adjacent genome locations, suggesting that their shared transcriptional background may be disadvantageous. Genes that are essential to mammalian survival and reproduction are less likely to occur in neighborhoods, although neighborhoods are enriched with genes that function in mitosis. We also found that gene orientation and protein-protein interactions are partially responsible for maintenance of gene neighborhoods.

**Conclusions:**

Our experiments using G-NEST confirm that tandem gene duplication is the primary driver of non-random gene order in mammalian genomes. Non-essentiality, co-functionality, gene orientation, and protein-protein interactions are additional forces that maintain gene neighborhoods, especially those formed by tandem duplicates. We expect G-NEST to be useful for other applications such as the identification of core regulatory modules, common transcriptional backgrounds, and chromatin domains. The software is available at http://docpollard.org/software.html

## Background

In every complete genome analyzed to date, the genomic locations of co-expressed genes have not been random. The clustering of co-expressed genes has been confirmed in the yeast [1-4], worm [1,2,5-8], fly [1,2,9,10], mouse [1,9,11-15], rat [1], cow [16], chimpanzee [17] and human [1,2,9,12-15,18-24] genome. Despite all of these studies, there is no consensus definition of a gene neighborhood with respect to size or content. Individual studies in worms and mice suggested that clusters contain 2–5 genes [7,25]. However, using a less conservative definition of clustering, clusters of 10–30 co-expressed genes covering 20–200 kb were identified in the *Drosophila* genome [11]. Significant long-distance co-expression has also been identified in yeast for gene pairs separated by up to 30 intervening genes or 100kb [26].

While most studies required that co-expressed genes be adjacent to each other to be called a cluster [9,12-14], other studies illustrated that non-adjacent pairs of genes as well as adjacent pairs of genes have correlated expression[26,27]. It is also possible that the distribution of neighborhoods depends on the biological context. For example, genes up-regulated in two cell types during replicative senescence are clustered, but those up-regulated during quiescence are not clustered [28]. Most recently, Weber and Hurst suggested that there are two primary types of gene neighborhoods in eukaryotes: type 1 clusters that are 2–3 genes in length and type 2 clusters that are much larger and contain functionally similar genes [29].

The causes of the gene neighborhood phenomenon—non-random gene order—remains a subject of considerable debate, especially in the genomes of multi-cellular eukaryotes. Tandem duplication is believed to be the primary driver of gene neighborhood formation [30], but there are many other potential drivers of neighborhood maintenance. In terms of mechanisms, neighboring genes may be co-expressed when they share the same open or closed chromatin conformation [4,31]. Also, adjacent genes co-oriented on the same strand (→, → or ←, ←) can both be transcribed when transcription fails to stop at the end of the first gene. This is called transcriptional read-through. Adjacent genes with a divergent orientation (←,→: opposite strands with adjacent start sites) share a bi-directional promoter, and thus, they may share cis-acting elements. At the level of function, there are several reasons why it might be advantageous for co-expressed genes to be co-localized. Co-functionality, whereby products of genes in the same cluster have common functions, has been suggested as a higher order organizing principle [32,33]. Gene neighborhoods may also be guided by “tissue-specificity”; genes that are expressed in the same tissue could be co-located in the genome. Essential genes—those that are required for the survival of the organism—may also have constraints on their genomic location [34]. In yeast, genes whose products interact are likely to be co-located [35]. In summary, possible causes of the gene neighborhood phenomenon include 1) tandem duplication, 2) shared chromatin domains, 3) transcriptional read-through, 4) shared cis-acting elements, 5) co-functionality, 6) tissue-specificity, 7) essentiality, and 8) protein-protein interactions. Some of these characteristics are inter-dependent.

Previous studies of these potential drivers of non-random gene order have been hampered by non-uniform analysis methods, sometimes resulting in paradoxical conclusions. In all transcriptome studies to date, definitions of what constitutes a cluster or gene neighborhood have been restricted to arbitrary rules such as requiring adjacency or a minimum number of genes or within a base pair region of fixed length or a minimum expression level. While some previous studies in prokaryotes have used sequence conservation in related species to identify gene neighborhoods [36-45], no studies of gene neighborhoods in eukaryotes have incorporated evolutionary sequence conservation.

In the current study, we developed a Gene Neighborhood Scoring Tool (G-NEST) and applied it to several large mammalian data sets. G-NEST combines genomic location, gene expression, and evolutionary sequence conservation data to score putative gene neighborhoods across all possible window sizes in terms of gene number or base pair length. The algorithm utilizes quantitative gene expression data, such as that derived from microarray or RNA-sequencing technologies. One of the key innovations of the G-NEST approach is that it scores the evolutionary conservation of gene neighborhoods using syntenic blocks. This feature enables the identification of neighborhoods containing paralogous, divergent, or unannotated genes. It also refines the requirement of adjacency used in many previous studies. Applying G-NEST to mammalian genomes, we find multiple explanations for the maintenance of non-random gene order.

## Results and discussion

### Overview of G-NEST

To identify gene neighborhoods with a high likelihood of biological significance, we developed a Gene Neighborhood Scoring Tool (G-NEST). The user provides G-NEST with the genomic locations and expression data for all genes in their data set. Included with the software release, G-NEST has syntenic blocks for ten mammalian organisms with human, mouse, and cow as reference genomes. However, a user has the option to upload their own syntenic blocks for their organisms of interest, which need not be mammalian.

**Figure 1:**
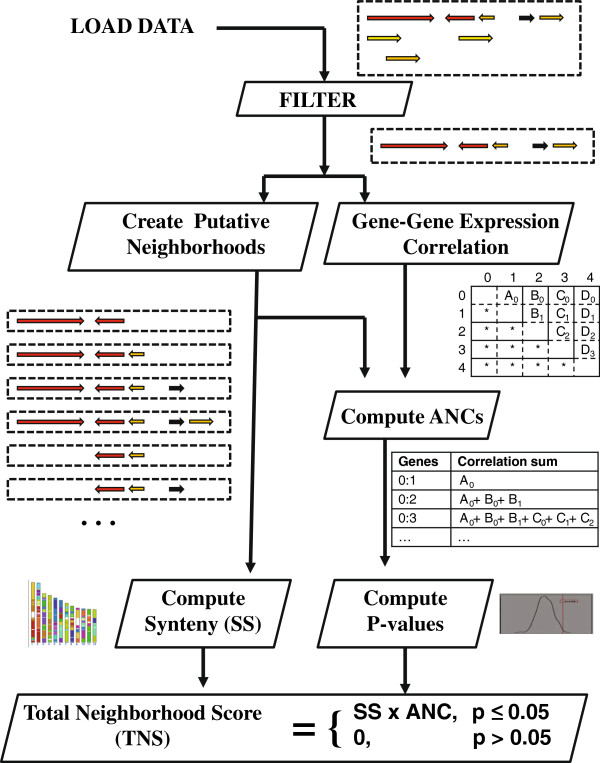
**Overview of G-NEST.** The user’s gene expression data is first filtered to remove overlapping transcripts. Next all possible gene neighborhoods are compiled based on the range, in number of genes and base pair width, of neighborhood sizes to test as requested by the user. Based on the user’s gene expression data, the correlation of every gene’s expression profile with the expression profile of every other gene in the genome is computed and stored in a matrix. Non-expressing genes, which are identified using user- supplied minimum gene expression level threshold or a minimum number of MAS5 detection calls, are assigned correlation values of 0. Given the genes within each potential neighborhood and the matrix of pairwise correlations, the Average Neighborhood Correlation is computed for each neighborhood. The ANC is the average of all pairwise correlations of genes in the neighborhood. For example, the ANC of a neighborhood with genes A, B, and C would be equal to [corr(A,B) + corr(B,C) + corr(A,C)] / 3. The significance of the observed ANC is then determined by comparing the ANCs computed from genomes with randomized gene order. Given the genes within each potential neighborhood and syntenic blocks for organisms of interest, a Synteny Score (SS) is computed as the proportion of genomes in which the synteny of the neighborhood is maintained. Finally, a Total Neighborhood Score (TNS) is computed from the Synteny Score (SS) and the Average Neighborhood Correlation (ANC).

After users upload their data, the data set is first filtered to remove transcripts that have overlapping genome coordinates (see Figure 1). When multiple transcripts overlap, the transcript with the highest gene expression is retained. Ties are broken by transcript length—longest wins. These non-overlapping genes, the “non-redundant” gene set, are then used to create all possible gene neighborhoods given the user’s defined range of possible neighborhood sizes in terms of gene number or base pair length. For example, if a user specifies neighborhood sizes of 2 to 4 genes, all possible neighborhoods of neighboring genes A, B, C, and D would be AB, ABC, ABCD, BC, BCD, and CD. We have experimented with neighborhood sizes of 2 to 50 genes and with 10 kb to 10 Mb.

The user’s gene expression data is used to compute the pairwise correlation (Spearman’s rho) of the expression level of every gene with every other non-redundant gene in the genome. We define a test statistic, the Average Neighborhood Correlation (ANC), which is the average of all pairwise expression correlations of all genes in the putative neighborhood. To determine the significance of each observed ANC, it is compared to the distribution of ANCs observed in randomized transcriptomes. To preserve the gene density and regional characteristics of the genome under study in our randomized null model, we retain the authentic positions of genes and shuffle their expression profiles randomly. P-values for each putative gene neighborhood on the chromosome are computed as the proportion of gene neighborhoods in the genome-wide null model (for that window size) with ANCs greater than the observed ANC.

For this study, the p-values are not adjusted for multiple hypotheses for several reasons. Our intent is to rank gene neighborhoods, not to make statements about the statistical significance of individual neighborhoods. The greater bandwidth of the un-adjusted p-value distribution provides a more meaningful ranking than does the much smaller bandwidth of the adjusted p-value distribution. Additionally, the observed ANC values are not independent, especially across window sizes, and the expected occurrence of true gene neighborhoods is not rare. These characteristics violate the assumptions of many commonly used p-value adjustment methods. Nonetheless, one may apply an appropriate multiple testing adjustment to the p-values computed by G-NEST if desired.

To determine whether a gene neighborhood in the reference genome is conserved in other genomes, a Synteny Score (SS) is computed for each neighborhood as a proportion, from 0 to 1, of genomes in which synteny of the neighborhood is maintained. For example, if syntenic blocks are provided for 9 species and the neighborhood in the reference genome maps to an unbroken segment of DNA in 7 of the 9 species, the synteny score (SS) would be 7/9 or 0.78. While ortholog mapping has been used successfully to determine neighborhood conservation in prokaryotes [36,37,41-46], it is less appropriate for mammalian genomes because the maps are incomplete, resulting in many neighborhoods being falsely identified as non-conserved. With the syntenic block method, a gene neighborhood resides within a span of base pairs on a chromosome and if these base pairs are syntenic with a span of base pairs in the second genome, then the neighborhood is considered “conserved” in the second genome.

Finally, for each putative neighborhood, a Total Neighborhood Score (TNS) is computed: TNS = (SS)(ANC) for *p ≤* 0.05 else 0, where SS (Synteny Score) is the proportion of genomes in which synteny is maintained, ANC (Average Neighborhood Correlation) is the average of all pairwise correlations of all genes in the neighborhood, and *p* is the p-value computed from randomized transcriptomes (i.e., the probability that the ANC is observed by chance). We evaluated various alternative TNS definitions by examining the number of non-expressed genes that fall within neighborhoods. Using the definition above, this is zero. The proposed definition appropriately demotes putative neighborhoods that contain non-expressed genes.

G-NEST automatically produces a full suite of graphs, genome browser custom tracks, text-based reports, and a database dump. The graphs provided include plots of the TNS, ANC, and p-value across all window sizes along each chromosome. Plots are produced for window sizes expressed as gene counts and as base pairs and with indices along the chromosome in gene positions and in base pairs. Custom tracks for the UCSC Genome Browser [47] (http://genome.ucsc.edu/) are automatically generated to visualize the TNS scores alongside other genomic information. G-NEST produces reports that include all information for each neighborhood and the best TNS associated with each gene. Finally, the database dump includes all input, intermediate, and output data created by G-NEST.

### Software availability

G-NEST is implemented as a LINUX command line program built on a PostgreSQL database. It was designed primarily to be used as a tool within a local Galaxy instance, but it can also be used as a stand-alone program. The software is available at http://docpollard.org/software.html and as Additional file 1 with an example in Additional file 2. It has been tested with both microarray and RNA sequencing data sets using a quad core 2.4 GHz processor and 8 GB RAM running Ubuntu Linux. G-NEST can also be run within Galaxy at http://korflab.ucdavis.edu/software.html.

### Application of G-NEST to microarray and RNA-Seq data sets

We applied G-NEST to several large publicly available mammalian data sets created using microarray and RNA sequencing technologies [48,49]. The data presented in this paper is primarily derived from an analysis of a microarray atlas of 61 mouse tissues, two replicates each, which we refer to as the “Microarray Atlas”. Additional results are presented using an RNA-Seq atlas of six human tissues—brain, cerebellum, heart, kidney, liver, and testis—which we refer to as the “Six-Tissue RNA-Seq Atlas”. For direct comparisons of microarray and RNA-Seq platforms, the results also include analyses based on a subset of the Microarray Atlas that includes only the same six tissues as in the Six-Tissue RNA-Seq Atlas: we refer to this as the “Six-Tissue Microarray Atlas”. Duplicate-Free versions of these data sets were produced as well. See “Data Set Selection” in Methods.

Eleven mammalian genomes—human, chimp, gorilla, orangutan, macaque, marmoset, mouse, rat, dog, horse, and cow—were used to generate syntenic blocks for these experiments (See “Generation of Syntenic Blocks”). For the mouse microarray data sets, syntenic blocks between the mouse and the other ten mammalian genomes were uploaded to G-NEST. For the human RNA-Seq data sets, syntenic blocks between the human and the remaining ten mammalian genomes were uploaded to G-NEST.

### Large gene neighborhoods are derived from smaller neighborhoods

**Figure 2:**
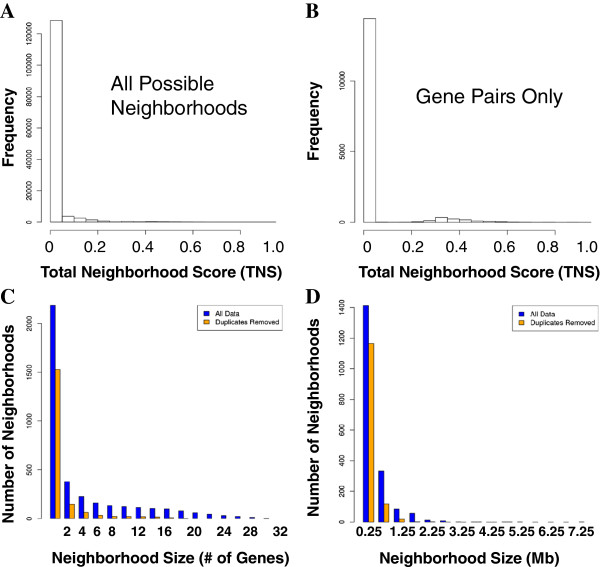
**Genome-wide Neighborhood Scores and Sizes. A-B**. Distribution of Total Neighborhood Scores (TNSs) computed from the Microarray Atlas data set including (**A**) all possible neighborhoods and (**B**) only possible neighborhoods with two genes. **C**-**D**. Distribution of neighborhood sizes (TNS > 0.2) on a (**C**) gene-wise and (**D**) base-pair-wise basis.

Using the Microarray Atlas, the genome-wide distribution of the Total Neighborhood Score (TNS) demonstrates that most genes are not in neighborhoods (TNS = 0), as expected, and that the TNS effectively distills thousands of possible neighborhoods down to a few high-scoring ones (see Figure 2A-B). While most high-scoring gene neighborhoods consist of only 2 or 3 genes, as observed previously [12], there are more than 1000 neighborhoods with more than 3 genes with TNS > 0.2. Some of these neighborhoods include as many as 30 genes (see Figure 2C). On a base-pair-wise basis, most neighborhood sizes are less than 1 Mb, but may be as high as 6–7 Mb (see Figure 2D). However, gene neighborhoods identified on a base-pair-wise basis appear to be heavily biased towards regions of low gene density and may include gene deserts. Therefore, the results presented in this paper are derived from window sizes based on gene counts. 

**Figure 3:**
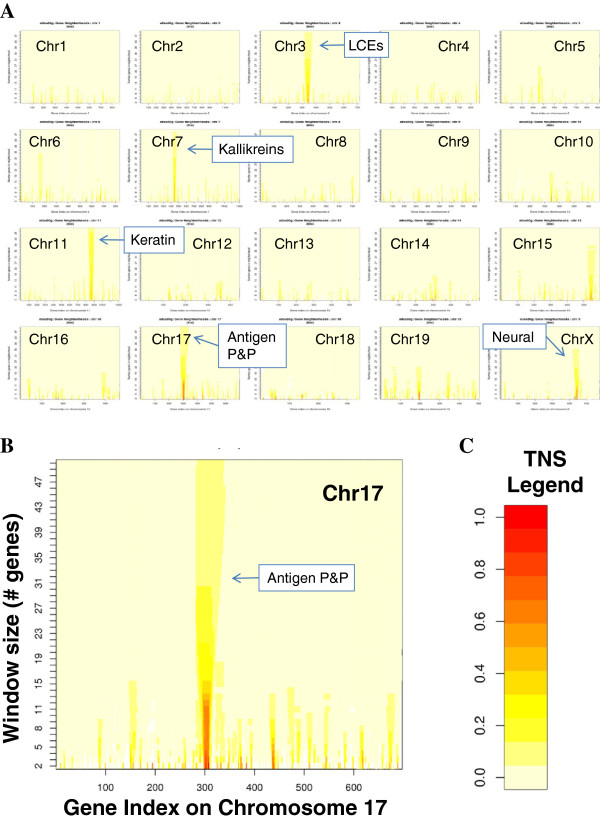
**Total Neighborhood Scores (TNSs) plotted across all window sizes on all chromosomes. A-B**. TNSs were computed using the Microarray Atlas data with window sizes from 2 to 50 genes. The x-axis represents the gene index which is the order in which the genes appear on the chromosome. The y-axis represents the window size from 2 to 50 genes. **A**. This birds-eye view shows all 20 chromosomes at a glance with the five largest high-scoring neighborhoods annotated (see text for full description). **B**. This TNS plot is a close-up of Chromosome 17 (from **A**). **C**. This legend shows the colors associated with TNSs in **A**-**B**.

Considering neighborhoods from 2 to 50 genes in length, large neighborhoods are primarily “shadows” of much smaller neighborhoods (see Figure 3). In other words, larger neighborhoods can appear high-scoring because they contain one or more high-scoring gene pairs with statistical significance persisting as the window size is expanded to include genes with poorly correlated expression. When considering neighborhoods on a base-pair-wise basis, the shadow effect of smaller neighborhoods persists. These findings are consistent with the assertion by Weber and Hurst that large clusters of correlated expression previously reported in *Drosophila melanogaster* may be a technological artifact [29]. Our results demonstrate that large co-expressed clusters of significance are extremely rare in mammalian genomes. 

**Figure 4:**
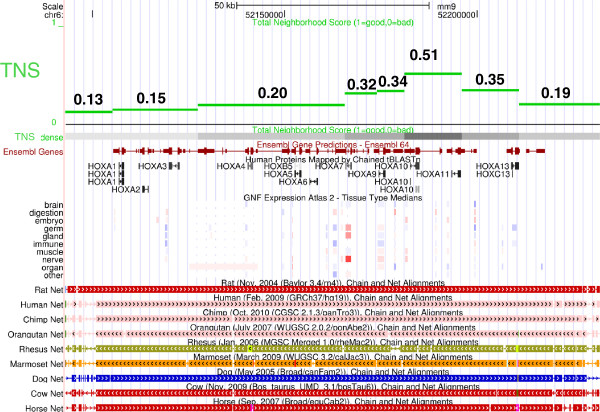
**The Hox cluster in the UCSC Genome Browser.** (http://genome.ucsc.edu/) A custom track of the TNS is displayed in full mode and in dense mode alongside the following publicly available tracks: predicted Ensembl genes, Human proteins mapped using tBLASTn, median gene expression from the Microarray Atlas data set, and chain/net tracks that indicate evolutionary conservation of genome sequence assembly with the genomes of other organisms. The TNS track was computed as follows: for each gene, the “best” associated TNS was identified and plotted. The “best” associated TNS is the maximum TNS of all gene neighborhoods that contain the gene. Note that gene expression is not well-correlated between members of the Hox cluster, while regional synteny is maintained in the mammalian genomes studied.

For a pragmatic approach to the “shadow” problem of smaller neighborhoods, users of G-NEST are offered the following suggestions. The scores (TNS values) should be viewed as a ranking of putative neighborhoods, rather than a binary “yes/no” designation of neighborhood. Larger putative neighborhoods should be explored in the UCSC Genome Browser using the “custom track” generated by G-NEST. An example of the TNS custom track, which shows the best/highest TNS at each genomic location, is shown in green in Figure 4. With this alignment of the TNS scores with the gene locations, biologists can readily determine which gene pairs are contributing the most to the overall TNS scores of the region. For example, in Figure 4, Hoxa10 and Hoxa11 must have well-correlated expression profiles. Biologists can incorporate any additional evidence they may have to determine whether the candidate locus highlighted by the high TNS score is worthy of further pursuit. For computational biologists who want to make use of genome-wide TNS scores, the TNS distribution for their data set of interest should be plotted to select an appropriate threshold score for their further analyses. Sufficiently high TNS thresholds will “cut out” the shadows while retaining the more highly co-expressed and co-conserved small neighborhoods.

### Highest scoring gene neighborhoods arose from tandem duplication

**Figure 5:**
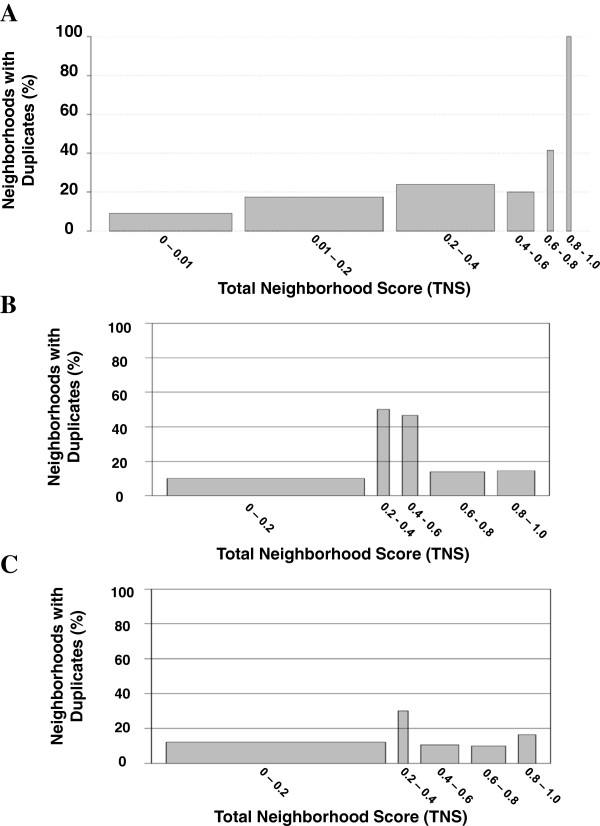
**Bar plots of the percentage of gene pairs that are duplicates, stratified by TNS.** The TNSs were computed using (**A**) all 61 tissues of the Microarray Atlas, (**B**) the six tissues of the Six-Tissue RNA-Seq Atlas, and (**C**) the same six tissues in the Six-Tissue Microarray Atlas. The height of each bar represents the percentage of neighborhoods with duplicates in the given range of TNSs. The width of each bar represents the total number of gene neighborhoods in the given range of TNSs.

A manual review of the highest scoring neighborhoods suggests that these neighborhoods were formed by gene duplications. To test this hypothesis genome-wide, we created a BLAST database of all protein sequences associated with the transcripts probed by the microarray and used the e-values from BLASTP results as an indicator of sequence similarity. When neighborhoods are stratified by TNS (see Figure 4), the mean e-values for high-scoring neighborhoods are significantly lower than for low-scoring neighborhoods (Wilcoxon rank sum (WRS) test; p < 2.6e-07) and their distributions are significantly different (Kolmogorov-Smirnov (K-S) test, p < 6.0e-08). Defining a gene duplication as a pair of genes with BLASTP e-value <1e-07 [10,29], nearly all of the high-scoring neighborhoods contain a gene duplication (see Figure 5). 

Interestingly, the results in Figure 5A also suggest that most gene duplications are not within neighborhoods (TNS < 0.2). Do most gene neighborhoods formed by tandem duplicates have a low TNS due to low expression correlation or low synteny? To answer this question, the ANCs and SSs of gene pairs that are duplicates were compared to gene pairs that are not duplicates. The ANCs of gene pairs that are duplicates are greater, on average, than the ANCs of gene pairs that are not duplicates (WRS p < 2.2e-16,). However, the SSs of tandem duplicates are lower, on average, compared to gene pairs that are not duplicates (WRS p < 2.2e-16). While 70% of adjacent gene pairs are syntenic across all 10 genomes relative to the mouse reference, only 40% of tandem duplicates are perfectly syntenic. Roughly 10-20% of all tandem duplicate gene pairs have an SS of zero or near zero and therefore probably arose from a recent duplication event. The remaining 30-40% of duplicate gene pairs may possibly be the result of ancient duplications that have become separated through genomic rearrangement. Further study is needed to determine whether both duplicates in each pair exist at the base of the Eutharian lineage. In summary, while tandem duplicates exhibit more highly correlated expression than other pairs, they are less likely to be linked across all mammalian genomes. It could be that, as suggested by Liao and Zhang, most co-expression of neighboring genes is disadvantageous [50] or that the de-coupling of duplicate gene pairs is somehow advantageous.

To determine if high co-expression, and thus, the high neighborhood scores, of tandem duplicates is an artifact of non-specific hybridization in the Microarray Atlas experiment, G-NEST was applied to the Six-Tissue RNA-Seq Atlas (see Methods). Neighborhoods (TNS > 0.2) were still enriched for gene duplications, although not as strongly as the Microarray Atlas (see Figure 5B). Gene neighborhood scoring of the Six-Tissue Microarray Atlas shows a similar profile of gene duplicate enrichment as the Six-Tissue RNA-Seq Atlas (see Figure 5C). Therefore, at least some of the duplicate phenomenon is not a technological artifact. In summary, our results confirm the observation in prior studies [51,52] that gene duplication is a substantial driver of gene neighborhood formation.

### Not all high-scoring neighbors are tandem duplicates

To determine whether high-scoring neighbors can occur in the absence of tandem duplication, G-NEST was applied to the Duplicate-Free Microarray Atlas data set. We found that even genes without shared ancestry can be co-located, co-expressed, and co-conserved. For example, the four highest-scoring gene pairs (all TNS > 0.7) are 1) Psmb9 and Tap1, 2) Rnf14 and Ndfip1, 3) Atp6ap1 and Gdi1, and 4) 1500032L24Rik and Ndufa6. Psmb9 and Tap1 (TNS = 0.76) are nearest neighbors in a divergent orientation with transcription start sites less than 500bp apart. Their co-expression and co-conservation is most likely due to a shared promoter region. Rnf14 and Ndfip1 (TNS = 0.76) are co-oriented and more than 100,000 bp apart. Their co-expression and co-conservation may be due, instead, to shared function; Ndfip1 activates E3 ubiquitin-protein ligases, Rnf14 is an E3 ubiquitin-protein ligase. Atp6ap1 and Gdi1 (TNS = 0.75) are co-oriented. Given that the transcription end of Atp6a1 and the transcription start of Gdi1 are only 300bp apart, it is likely that these genes are co-expressed due to transcriptional read-through or shared chromatin domains. They also have similar functions in that Atp6ap1 has ATPase activity and Gdi1 regulates GTPase activity. Finally, 1500032L24Rik and Ndufa6 (TNS = 0.73) are convergently oriented. Together, these two genes span 8Kb. They may be co-located due to shared function or shared ancient origins as they are both mitochondrial proteins. In summary, gene neighborhoods can be formed by factors other than tandem duplication.

### Largest gene neighborhoods also arose from tandem duplication

We identified five non-redundant large gene neighborhoods (10 or more genes) with TNS > 0.3 using the Microarray Atlas, one on each of mouse chromosomes 3, 7, 11, 17, and X (see Figure 3). Each of these highest scoring large neighborhoods contains gene duplications. The neighborhood on chromosome 3, annotated as “LCEs” in Figure 3, contains a large cluster of late cornified envelope genes that are expressed mainly in external epithelia such as the skin. The neighborhood on chromosome 7 contains a large cluster of kallikreins that are all highly expressed in the mouse thyroid gland (see “Kallikreins”, Figure 3). The chromosome 17 neighborhood, annotated as “Antigen P&P” in Figure 3, contains at least three different sets of duplicate genes—antigen peptide transporters 1 and 2, proteasome subunit beta types 8 and 9, and histocompatibility class II antigens—all of which appear to function in antigen processing and presentation (see “Antigen P&P”, Figure 3). The neighborhood on chromosome 11 contains more than 10 and possibly as many as 50 keratin-associated genes (see “Keratin-associated”, Figure 3). These genes are most highly expressed in digits, snout epidermis, and tongue. Finally, the neighborhood on chromosome X (see “Neural”, Figure 3) consists of genes most highly expressed in neural tissue and is formed by at least three different gene duplications: Bex1 and Bex2 are duplicates with Ngfrap1 highly similar (BLASTP e-value 8x10^-7^); Gprasp1, Gprasp2, and Bh1hb9 are duplicates; Arxes1 and Arxes2 are duplicates. The fact that all of the largest high-scoring gene neighborhoods in this data set contain tandem gene duplicates underscores the importance of gene duplication to the phenomenon of non-random gene order. Our results suggest that large, co-expressed, conserved neighborhoods of genes are extremely rare in mammalian genomes, and that in the few cases where they occur, they are the result of tandem duplication.

### The Hox cluster and other large neighborhoods of interest

Perhaps the most well-known gene neighborhood is the *Hox* gene cluster. It is not among the largest, highest scoring neighborhoods, because gene expression among members of this cluster is not as well-correlated. However, the TNS within the Hox cluster does rise as high as 0.51 due to the gene expression correlation of *Hoxa10* and *Hoxa11* combined with the fact that the entire locus is well conserved (see Figure 4). Genome-wide analyses reveal that there are on the order of 100 neighborhoods with statistical significance equal to the *Hox* gene cluster. It could be expected that many of these neighborhoods are biologically significant and worthy of further exploration. A complete list of all putative neighborhoods, their locations, and scores based on the Microarray Atlas are provided in Additional file 3. See Additional file 4 for a UCSC Genome Browser custom track of the best associated TNSs.

To identify large neighborhoods that were not formed by tandem duplication, we reviewed the results of running G-NEST on the Duplicate-Free Microarray Atlas. Looking at neighborhoods of 10 genes, there were three non-redundant neighborhoods with TNS > 0.2: one each on chromosomes 3, 5, and 7. The neighborhood on chr3 is made up of LCE genes as described previously (see “LCEs” Figure 3). These genes have related function, but are not homologous (BLASTP e-value < 0.2). The neighborhood on chr5 contains the casein genes: Csn1S1, Csn2, Csn1s2a, Csn2b, and Csn3. The caseins are milk proteins that are an essential component of mammalian milk. The neighborhood on chr7 includes proline-rich proteins such as SCAF1, IRF3, PRR12, PRRG2, BCL2L12. Proline-rich proteins are typically intrinsically unstructured; that is, they lack a stable tertiary structure. This neighborhood contains secretory proteins that are expressed in the brain, by skin, by salivary gland, and so forth. Curiously, the caseins in the neighborhood on chr5 also lack stable tertiary structure and are secreted by the mammary gland. The casein gene neighborhood is well-known and well-studied [53-55]. That we find it among high-scoring neighborhoods is a further proof of concept for G-NEST. The chr7 neighborhood (approximately chr7:52,253,000-52,416,000 in the NCBI37/mm9 assembly), which is most coordinately expressed in the brain, may represent a novel gene neighborhood of biological interest.

### Genes within high-scoring neighborhoods are not broadly expressed

Previous studies, which excluded tandem duplicates, have suggested that large gene neighborhoods are comprised of broadly expressed “housekeeping” genes [56]. To determine the “expression breadth” of each gene in our experiments, we computed Tau, a measure of tissue specificity, as described by Yanai et al. [57]. Tau incorporates the number of samples in which a gene is expressed, as well as the level of expression. For N samples, a gene with expression in only one sample would have a Tau = N-1. A gene that is expressed equally in all samples would have a Tau = 0.

**Figure 6:**
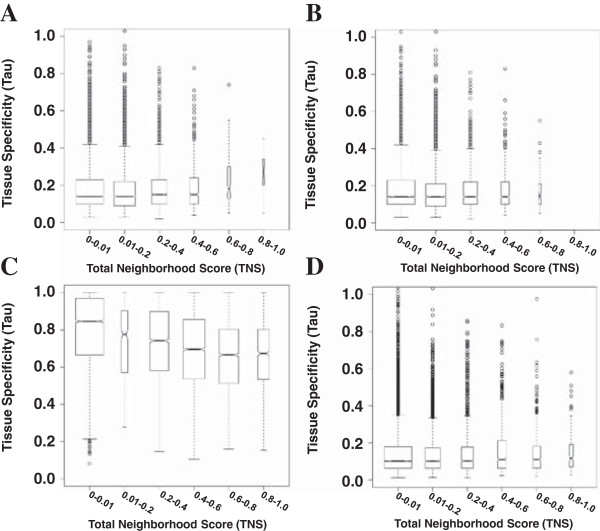
**Box plots of tissue specificity, stratified by best TNS.** The distribution of Tau (a measure of tissue specificity), stratified by best associated TNS is shown based on (**A**) the 61 tissues of the Microarray Atlas, (**B**) the Duplicate-Free Microarray Atlas, (**C**) the Six-Tissue RNA-Seq Atlas, and (**D**) the Six-Tissue Microarray Atlas. The width of each box plot represents the total number of genes with best associated TNS in the given range of TNSs. The whiskers of each boxplot denote the range of the data, the circles mark outliers, and the boxes mark the quartiles above and below the median (center line). These boxplots are notched; when two boxes' notches do not overlap, this is strong evidence of significantly different medians.

Our analysis of the Microarray Atlas suggests that the most highly scoring neighborhoods have higher tissue-specificity (see Figure 6A). However, analysis of the Duplicate-Free Microarray Atlas suggests that this pattern of tissue-specific expression is driven primarily by duplicated genes (see Figure 6B). Using the Six-Tissue RNA-Seq Atlas, we found lower tissue-specificity for high-scoring neighborhoods (see Figure 6C) while the Six-Tissue Microarray Atlas showed unchanged tissue-specificity (see Figure 6D). Six tissues may not be a sufficient number for measuring tissue specificity. Lercher et al. identified house-keeping gene clusters in the human genome using 14 tissues [56]; however, the breadth of expression was determined by presence or absence of the transcript, rather than a measure of quantitative abundance. It will be useful to revisit the tissue-specificity of gene neighborhoods as larger atlases of RNA-Seq data become available. 

### Genes within high-scoring neighborhoods are more highly and more variably expressed

**Figure 7:**
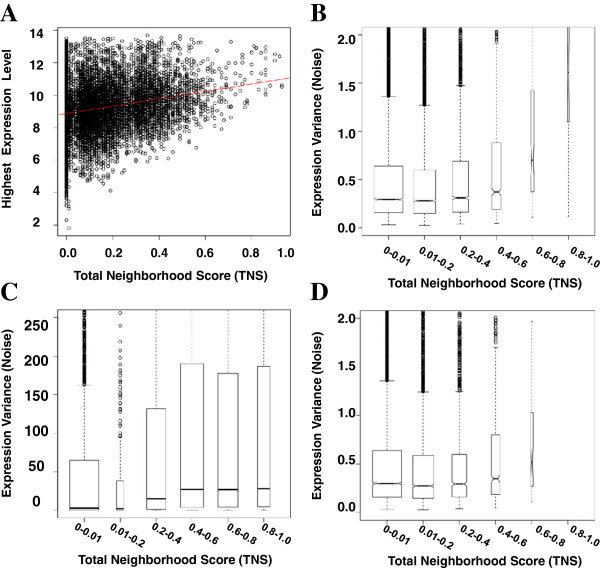
**Genes within high-scoring neighborhoods are more highly and variably expressed.** (**A**) The TNS and expression intensities in this scatterplot are based on the 61 tissues of the Microarray Atlas. Red line denotes best linear fit. (**B**-**D**) Boxplots of gene expression variances stratified by TNS are computed for (**B**) the Microarray Atlas, (**C**) the Six-Tissue RNA-Seq Atlas, and (**D**) the Duplicate-Free Microarray Atlas. The width of each box plot represents the total number of genes with best associated TNS in the given range of TNSs. The whiskers of each boxplot denote the range of the data, the circles mark outliers, and the boxes mark the quartiles above and below the median (center line).

In order to investigate whether genes in high-scoring neighborhoods have unique expression patterns, we computed maximum TNS (over all neighborhoods containing the gene), maximum gene expression intensity (across all tissues), and the variance of gene expression intensity (across all tissues) for each gene, exclusive of silent genes (See “Identification and Processing of Silent Genes” in Methods). As shown in Figure 7A, genes in higher scoring neighborhoods exhibit higher maximal gene expression. Genes within neighborhoods (TNS > 0.2) have a higher maximum gene expression than other genes (TNS < 0.2) (WRS p < 2.2e-16). Higher-scoring neighborhoods also contain genes with more variable (noisier) gene expression, on average, than lower-scoring neighborhoods (WRS p = 1.12e-11, see Figure 7B-D), independent of microarray or RNA-Seq assay (Figure 7B-C) or even when duplicate genes are removed (Figure 7D). Despite the differences in dynamic range achievable with the microarray and RNA-Seq platforms, the observation that gene neighborhoods contain genes with “noisier” expression appears to be technology-independent.

To determine whether the higher neighborhood scores associated with genes more highly and variably expressed across tissues is due to gene expression correlation or to evolutionary sequence conservation, the best ANC and best SS associated with each gene were also calculated in relation to expression intensity and variance. Best ANC and SS values are both associated with higher maximal gene expression intensity (ANC: WRS p < 2.2e-16, K-S p = 1.9e-14; SS: WRS p = 2.2e-12, K-S p = 9.8e-10). However, only the best ANC value is associated with higher expression variance (WRS p = 1.3e-15). On average, genes with higher variance have lower synteny scores (WRS p = 1.6e-04). Therefore, highly expressed genes are more likely to be in high-scoring neighborhoods because they have more highly correlated expression with neighboring genes and a higher degree of evolutionary conservation. Noisy genes—those more variably expressed—are more likely to be in high-scoring neighborhoods only due to higher expression correlation with neighbors. Noisy gene pairs do not have evolutionarily conserved synteny.

The association of noisy gene pairs with poorer evolutionary conservation suggests that the transcriptional noise is somehow disadvantageous. It is possible that the transcription of the neighboring genes interferes with one another. Our results are consistent with the hypothesis of Liao and Zhang that transcriptional interference is potentially sub-optimal [50].

### Gene neighbors whose protein products interact are primarily those that arose through gene duplication

To determine whether mouse genes with interacting products are more likely to occur in the same neighborhood, we first collected all gene pairs that did and did not interact at the protein level and compared the TNS distributions of these two sets of gene pairs. The TNSs of gene pairs which interact at the protein level are significantly greater than the TNSs of gene pairs with no interactions (WRS p = 1.0e-13, K-S p = 4.0e-4). In fact, there were significantly more interactions between proteins derived from same-neighborhood genes for all neighborhood sizes tested (up to 10 genes), although these significant tests were driven by no more than three interacting proteins in each neighborhood.

Three neighborhoods were identified with three interactions each. The first neighborhood is comprised of three members of the STAT (Signal transducer and activator of transcription) family: *Stat5a*, *Stat3*, and *Stat5b*. These three genes encode transcription factors that, when phosphorylated, dimerize and translocate to the nucleus where they activate transcription. Coordinated regulation of this gene neighborhood may further enable the heterodimerization of Stat3:Stat5 or Stat5a:Stat5b. The second neighborhood is comprised of cell adhesion proteins: the cadherins *Cdh6, Cdh9*, and *Cdh10*. They are in a large neighborhood of 6 Mb in size and their BLASTP e-value of 0 suggests that they are duplicates. Coordinated regulation of this gene neighborhood could potentially be advantageous for the maintenance of cell positional stability and communication. The third neighborhood is a small neighborhood of only 43 kb that contains *CD3G, CD3D*, and *CD3E*. These encode the gamma, delta, and epsilon chains of the T-cell surface glycoprotein CD3 which associates with the T-cell receptor (TCR) to activate T lymphocytes. Coordinated regulation of this gene neighborhood could be expected to facilitate the formation of the TCR protein complex. In summary, the functional advantages of coordinated expression may be the evolutionary force that maintains neighborhoods of genes whose protein products interact.

Given that all three of these neighborhoods with three interactions contain tandem duplicates, the analysis was repeated with the Duplicate-Free Microarray Set. Without duplicates, the TNSs of neighbors whose proteins interact were not significantly different from the TNSs of non-interacting neighbors. Reviewing all duplicate-free gene pairs with TNS > 0.2, only one interacting pair was identified: neighbors Sec24a (protein transport protein Sec24a) and Sar1b (GTP-binding protein Sar1b) are not homologous and have a high neighborhood score (TNS = 0.58). These neighboring genes are divergently oriented with transcription start sites that are less than 10kb apart, likely sharing a promoter region. Overall, our results suggest that gene neighbors whose protein products interact are primarily those that arose through gene duplication. Therefore, protein interaction is unlikely to be a driver of gene neighborhood formation, but may play a role in neighborhood maintenance, especially for those neighborhoods arising as a result of tandem duplication.

### Gene neighborhood maintenance is not independent of gene orientation

Non-overlapping adjacent gene pairs can exist in one of three possible orientations: 1) both on the same strand (→, → or ←, ←), 2) on different strands with divergent transcription (←, →), or 3) on different strands with convergent transcription (→, ←). Previous studies have indicated differential occurrence of these orientations among adjacent co-expressed gene pairs [12,58]. To determine whether gene pairs within high-scoring neighborhoods are enriched for any particular orientation, the TNS distributions for gene pairs with each orientation were compared using the Microarray Atlas. The mean TNS is greater among co-oriented pairs compared to convergent or divergent pairs (WRS p = 1.3e-03 and p = 8.4e-04, respectively). The same is true of the mean ANC (WRS p = 2.3e-05, p = 8.1e-08). The mean SS is lower among co-oriented pairs compared to divergent pairs (p = 3.6e-03), but not convergent pairs. However, when the same tests are applied to the G-NEST results from the Duplicate-Free Microarray Atlas, neither the TNS nor the SS significantly differs by orientation. Only the ANC is significantly different: the mean ANC is greater among co-oriented pairs compared to divergent pairs (p = 5.1e-03), but not convergent pairs.

The fact that neighborhood scores, but not synteny scores, are greater among co-oriented pairs compared to other orientations suggests that transcriptional read-through may occur but is generally disadvantageous. Furthermore, the different results obtained when duplicates are removed suggest that much of this observation is driven by tandem duplication.

### Essential genes are more likely to be isolated

Essential genes are the minimum set of genes required for organism survival. Liao and Zhang defined essential genes in the mouse genome as those genes whose deletion results in lethality before reproduction or in sterility [59]. We used their method [50] to determine essential genes: those with phenotypic annotations of embryonic lethality (MP:0002080), prenatal lethality (MP:0002081), survival postnatal lethality (MP: 002082), premature death (MP: 0002083), or an abnormal reproductive system (MP:002160, MP:0001919) were deemed “essential”. Using the Microarray Atlas, the maximum TNS associated with an essential gene is slightly, but statistically significantly lower, on average, than that of genes with other phenotypic annotations (WRS p < 4.4e-05). The TNS distributions of essential and non-essential genes are significantly different (K-S p < 1.1e-06). The results hold when the experiment is repeated using the Six-Tissue RNA-Seq Atlas and the 120 human essential genes identified by Liao and Zhang [50] (WRS, p < 0.03, K-S p < 0.022). It is likely that the lower statistical significance of the experiment with the Six-Tissue RNA-Seq Atlas is due to the much smaller set of known human essential genes. Surprisingly, even when duplicates are removed (Duplicate-Free Microarray Atlas), the maximum TNS associated with an essential gene is slightly lower, on average, than non-essential genes (WRS p < 2.175e-06) and the TNS distributions of essential and non-essential genes significantly differ (K-S p < 2.751e-05).

It could be expected that essential genes would be more vulnerable to perturbations in their expression. Given that genes in the same neighborhood share a transcriptional background and often influence each other’s expression [8], the expression of any one gene within a neighborhood can be sub-optimal [50]. Therefore, the isolation of essential genes may reflect their need to maintain reliable and stable gene expression.

### Gene neighborhoods are enriched with genes involved in mitosis

To determine whether genes within high-scoring neighborhoods are enriched with any particular functions, gene set enrichment analysis (GSEA) [60,61] was applied to all mouse genes ranked by their maximum TNS (over all neighborhoods containing the gene) based on the Microarray Atlas data. GSEA enables the user to look for functional enrichment of gene ontology (GO) annotations within neighborhoods without choosing an arbitrary cut-off of the TNS. When only large gene sets (100 or more genes associated with the GO term) are considered, genes within high-scoring neighborhoods are enriched for the GO term, “mitosis” (FWER p < 0.05). Repeating the analysis using the Duplicate-Free Microarray Atlas data, genes within high-scoring neighborhoods were enriched for the GO term, “cell division” (FWER p < 0.05). Therefore, the enrichment of gene neighborhoods with genes involved in mitosis appears to be true even of those neighborhoods not formed by tandem duplicates.

Mitosis—the division of nuclear chromosomes into two identical sets—is a process that is fundamental to eukaryotic life. Intuitively, genes involved in cell division seem like they would be broadly expressed. However, a manual review of mitosis-related genes with highest TNS, such as *Birc5* and *Nedd1*, revealed tissue-specific expression. That these genes occur within neighborhoods in mammalian genomes suggests that clustering into neighborhoods of a common transcriptional background may be highly advantageous to their coordinated regulation.

### Future applications

While we have demonstrated G-NEST using gene expression data from mammalian tissue samples, gene neighborhood scoring with G-NEST has numerous other potential applications. G-NEST can be used with other types of biological experiments, such as extended time courses and treatment comparisons. In addition, results from many experiments can be combined, because G-NEST is platform-agnostic. Liao and Zhang posited that core regulatory modules can be identified by seeking conserved gene neighborhoods [50]. Indeed, G-NEST could be used for this purpose.

Given the petabytes of information now aligned with genome sequence, the scores produced by G-NEST can be uploaded to the Genome Browser and visualized in the context of this data. For example, Total Neighborhood Scores can be intersected with histone marker peaks, DNA hypersensitivity sites, DNA methylation, transcription factor binding sites, phenotype associations, and structural variations such as SNPs, indels, and copy number variations to better understand the factors contributing to a gene’s transcriptional background and the potential effects of genetic variation. G-NEST can also be used to identify gene neighborhoods important to a particular biological state and, when intersected with epigenetic information, to determine the effective size of chromatin domains.

Broadly, G-NEST is sufficiently flexible to be useful for correlation of features other than gene expression. The gene expression table uploaded to G-NEST could be a table of any other measurement that can be distilled down to a single value per gene and sample. Given that the code is open source, it is even possible to try out other definitions of gene neighborhoods.

## Conclusions

While demonstrating a gene neighborhood scoring technique, we investigated numerous potential contributors of non-random gene order in mammalian genomes: 1) gene orientation, which exerts its effects through characteristics such as transcriptional read-through and shared cis-acting elements, 2) co-functionality, 3) tissue-specificity, 4) expression intensity and variance, 5) essentiality, 6) protein-protein interactions, and 7) tandem duplication. The highest scoring and largest neighborhoods are formed by tandem gene duplication. Furthermore, we find some evidence for maintenance of these gene neighborhoods by co-functionality and non-essentiality, and among neighborhoods formed by tandem duplicates, by favorable gene orientation and protein-protein interactions. These phenomena were brought to light by using a flexible definition of gene neighborhoods, learning neighborhood size from the data, and quantitatively scoring expression correlation and evolutionary sequence conservation within neighborhoods to highlight the strongest clusters of co-expressed, conserved genes.

As the volume of genome data grows, G-NEST will be a useful tool for integrating and interpreting diverse data types. Built to run as a Galaxy tool and as a stand-alone program, it is intended to be accessible to both biologists and bioinformaticians. We expect that the Total Neighborhood Score (TNS), when paired with other genomic, epigenomic, and transcriptomic data, will shed light on regulatory processes that exceed the domain of a single gene.

## Methods

### Data set selection

The “Microarray Atlas” data set which contains gene expression intensity estimates from two replicates of each of 61 mouse tissues [48] was downloaded from NCBI GEO (GSE1133, PMID: 15075390). The “Six-Tissue RNA-Seq Atlas” data set which contains gene expression estimates from six human tissues—brain, cerebellum, heart, kidney, liver, and testis— was downloaded from the authors’ supplementary materials [49]. The “Six-Tissue Microarray Atlas” is the six mouse tissue subset (brain, cerebellum, heart, kidney, liver, and testis) of the Microarray Atlas data set that corresponds to the same six human tissues in the Six-Tissue RNA-Seq Atlas. To generate the duplicate-free data sets, protein sequences for the BLAST database were downloaded from Ensembl Release 57 [62] for the human “Duplicate-Free Six-Tissue RNA-Seq” data set and Release 52 for the mouse “Duplicate-Free Microarray Atlas” data set. Duplicates were defined as a pair of genes whose amino acid sequences have a BLAST e-value < 1e-07, as used in prior studies of gene neighborhoods in higher eukaryotes [10,29]. One of the genes in each duplicate pair was removed in each duplicate-free data set.

### Microarray data pre-processing

Each probe on the chip was remapped to an Ensembl transcript using methods described by Dai et al. [5]. In short, the custom chip definition file follows these rules: (1) A probe must hit only one genomic location, (2) Probes that can be mapped to the same target sequence in the correct direction are grouped together in the same probe set, and (3) Each probe set must contain at least three oligonucleotide probes and probes in a set are ordered according to their location in the corresponding exon. Genome locations for these transcripts were downloaded from the Ensembl database, release 52 [62].

Gene expression values were obtained by pre-processing the data sets with a customized set of pre-processing algorithms in R/Bioconductor [63]: background correction “mas”, normalization algorithm “invariantset”, perfect match correction algorithm “mas”, and summary algorithm “liwong”. Harr and Schlotterer evaluated 54 combinations of background correction, normalization, perfect match correction, and summary algorithms and determined that the above four selections yielded the highest correlation coefficient for the identification of co-regulated genes in known bacterial operons [64]. While the popular pre-processing methods improve the consistency of gene expression levels across chips, algorithms that accurately predict the actual gene expression level are more favorable to detect co-regulated genes [64]. After these correction and normalization, and summary steps, all expression values were log transformed (base 2).

### Identification and processing of silent genes

Transcripts that are not expressed may have gene expression profiles that correlate well with each other. It is not appropriate to discard these transcripts when searching for gene neighborhoods because the fact that they are not expressed is important information. We experimented with several strategies to handle non-expressed transcripts. Based on manual inspection of the resulting gene neighborhoods, we found that the most appropriate strategy was to set all pairwise correlations to zero for any transcript which is not expressed in a minimum number of samples. In G-NEST, these are termed “silent genes”. For the Microarray Atlas data, probes on the microarray for which there were not at least 12 “Present” MAS5 detection calls across the 122 arrays were deemed “silent”. For the Six-Tissue RNA-Seq Atlas, the filter was set to 0.2. In other words, silent genes were those genes with maximum expression level < 0.2 RPKM.

### Generation of syntenic blocks

Neighborhood scoring with G-NEST is partially dependent upon evolutionary sequence conservation at the neighborhood level. In other words, are the neighboring genes in the reference genome (“mouse” for the Microarray Atlas data) also neighboring in other mammalian genomes? Ortholog maps (i.e. gene from genome 1 = gene from genome2) between genomes are incomplete and so most putative gene neighborhoods in mammalian genomes would be considered “not conserved” merely due to a missing ortholog in the map. We instead use the concept of synteny: a gene neighborhood resides within a chromosomal location (span of base pairs) and if these base pairs are syntenic with a span of base pairs in the second genome, one could say that the neighborhood is conserved in the second genome.

The determination of whether or not a chromosomal location is syntenic in other genomes is dependent upon the desired resolution. Within a span of base pairs, the region may mostly be syntenic, but there may be small alignments--alignments within intergenic DNA, local inversions, or other variations--within the neighborhood that are not syntenic, but are inconsequential to the gene neighborhood.

To determine optimal parameter selection for synteny at the neighborhood level, we manually assigned neighborhood-level mouse-human, mouse-cow, and mouse-opossum synteny designations for 150 putative neighborhoods of less than 1 Mb in size on mouse chromosome 5. For manual inspection, we used the chain and net tracks on the UCSC Genome Browser (assemblies NCBI37/mm9, Baylor4.0/bosTau4, and Broad/monDom5) [65,66] that show alignments between different genomes so a user can visualize, at a base pair resolution, which pieces of one genome align to another. These manually derived designations of synteny at the neighborhood level were then used to test a broad range of parameters for the determination of syntenic blocks using Cinteny [67].

Markers from all high-coverage mammalian genomes—human, chimp, gorilla, orangutan, macaque, marmoset, mouse, rat, dog, horse, and cow—were uploaded to the Cinteny server (http://cinteny.cchmc.org/). Software, written in Perl and R, was developed to automatically download syntenic blocks from Cinteny for a broad sweep of parameter choices and to compute false positive and false negative rates. Compared with the manual designations, the automated designations using syntenic blocks agreed, at best, 97.3%, 76.7%, and 82.0% of the time for the human, cow, and opossum genome comparisons with the mouse genome, respectively. It should be noted that the manually assigned synteny designations are imperfect due to ambiguities. The high score for the mouse-human comparisons suggests that with a high-quality genome sequence, gene neighborhood synteny can be accurately determined using this method.

Cinteny’s parameters include minBlk, maxGap, and numMark. The minBlk parameter (in kb) is the minimum size of the smallest syntenic block. The maxGap parameter (also in kb) is the maximum gap between two syntenic blocks that can be aggregated into one large syntenic block. The number of markers, numMark, refers to the minimum number of markers required to define a syntenic block.

A “false negative” occurs when the automated syntenic block method determines that a gene neighborhood is not syntenic, when, in fact, the neighborhood is syntenic. A “false positive” occurs when the automated syntenic block method determines that a gene neighborhood is syntenic when it is not. The effect of minBlk and of numMark on either type of error is negligible, even for distant genomes such as mouse and opossum (see Additional file 5: Figure S2). However, both types of errors—false positives and false negatives—are impacted by the setting of the maxGap parameter (see Additional file 5: Figures S2). Overall accuracy of gene neighborhood synteny detection continues to improve with increasing maxGap as the dramatic improvement in the false negative rate outweighs the relatively small increase in false positives (Additional file 5: Figure S3). We also explored MaxGap settings larger than 1 Mb. For the mouse-opossum comparison, the percent accuracy dropped dramatically at MaxGap > 2.5 Mb (Additional file 5: Figure S4). Based on these experiments, we used the following parameter settings MaxGap = 1 Mb, MinBlk = 100 kb, and NumMark = 2 to generate syntenic blocks that were used as input to G-NEST for the results presented in this paper.

### Algorithmic optimizations

G-NEST is optimized for speed and low memory usage. Key optimizations include a) a customized calculation of the Spearman correlation that extracts the constant and reduces the number of arithmetic computations when generating the pairwise correlation matrix, b) routines that leverage the between- and within-window size redundancies to reduce the number of computations when computing ANC values, and c) use of PostgreSQL database to leverage the power of indexing to increase memory access speeds and reduce the physical memory requirements.

### Statistical analyses

A Wilcoxon rank sum (WRS) test with continuity correction (also known as a Mann–Whitney U) from the R programming language was used to determine if the mean of the TNS (or ANC or SS) distribution differed between gene sets of interest (e.g. high expressing vs low expressing genes). A two-sample Kolmogorov-Smirnov (K-S) test was used to determine if the TNS (or ANC or SS) observations associated with a gene set are drawn from the same distribution as another gene set. For both statistical tests, significance was determined by a p-value less than or equal to 0.05.

### Function enrichment analysis

Gene ontology (GO) annotations were downloaded from Ensembl [62]. In-house scripts were written to convert these annotations into custom gene sets for use with GSEA [60,61]. Genes were ranked by best associated TNS score. The enrichment of functions associated with genes towards the top and bottom of this ranked gene list were assessed using the “GseaPreranked” tool within GSEA. Significance was determined by the stringent family-wise error (FWER) multiple testing correction p-value of less than or equal to 0.05.

## Abbreviations

ANC: Average Neighborhood Correlation; BLAST: Basic Local Alignment Search Tool; BLASTP: Protein BLAST; G-NEST: Gene Neighborhood Scoring Tool; GO: Gene Ontology; GSEA: Gene Set Enrichment Analysis; K-S: Kolmogorov-Smirnov; SS: Synteny Score; TNS: Total Neighborhood Score; WRS: Wilcoxon rank sum.

## Competing interests

The authors declare that they have no competing interests.

## Authors’ contributions

DGL conceived of the study, participated in its design, implemented and executed all experiments, interpreted results, and drafted the manuscript. WFM architected and implemented the final version of G-NEST. ASH architected and implemented an intermediate version of G-NEST and provided technical assistance. MR participated in experimental design, interpreted results, and helped to draft the manuscript. JBG interpreted results. IK provided guidance on Galaxy implementation of G-NEST and access to computing resources. KSP participated in study design, analyzed and interpreted results, and helped to draft the manuscript. All authors read and approved the final manuscript.

## Supplementary Material

Additional file 1 **G-NEST software.** The Gene NEighborhood Scoring Tool (G-NEST) combines genomic location, gene expression, and evolutionary sequence conservation data to score putative gene neighborhoods across all window sizes. See README file for installation instructions. Note: the software requires PostgreSQL 9.0 or above. Click here for file

Additional file 2 **G-NEST Examples.** Example data files for the Gene Neighborhood Scoring Tool. Click here for file

Additional file 3 **Report file of all possible gene neighborhoods.** This text file contains all information associated with each gene neighborhood (genomic location, genes, neighborhood size, ANC, p-value, TNS, synteny, etc.). Scores were computed based on the Microarray Atlas. Genome coordinates are for assembly mm9 of the mouse genome. Click here for file

Additional file 4 **TNS custom track for UCSC Genome Browser.** This custom track can be uploaded, in its compressed form, to the UCSC Genome Browser (http://genome.ucsc.edu/) to view the best TNS associated with each gene in the mouse genome. The “best” TNS is the maximum TNS of all gene neighborhoods that contain the gene. TNSs were computed based on the Microarray Atlas. Click here for file

Additional file 5 Supplementary Figures. Click here for file
