# Supplementary material for: G-NEST: a gene neighborhood scoring tool to identify co-conserved, co-expressed genes
Source: BMC Bioinformatics. 2012 Sep 28;13:253. doi: 10.1186/1471-2105-13-253 (PMC3575404; doi:10.1186/1471-2105-13-253)
Supplement: Additional file 5 — Supplementary Figures. [file 1471-2105-13-253-S5.pdf]

Additional data file from “GNEST: a gene neighborhood scoring tool to identify co-conserved, co-expressed genes” Lemay et al, 2012

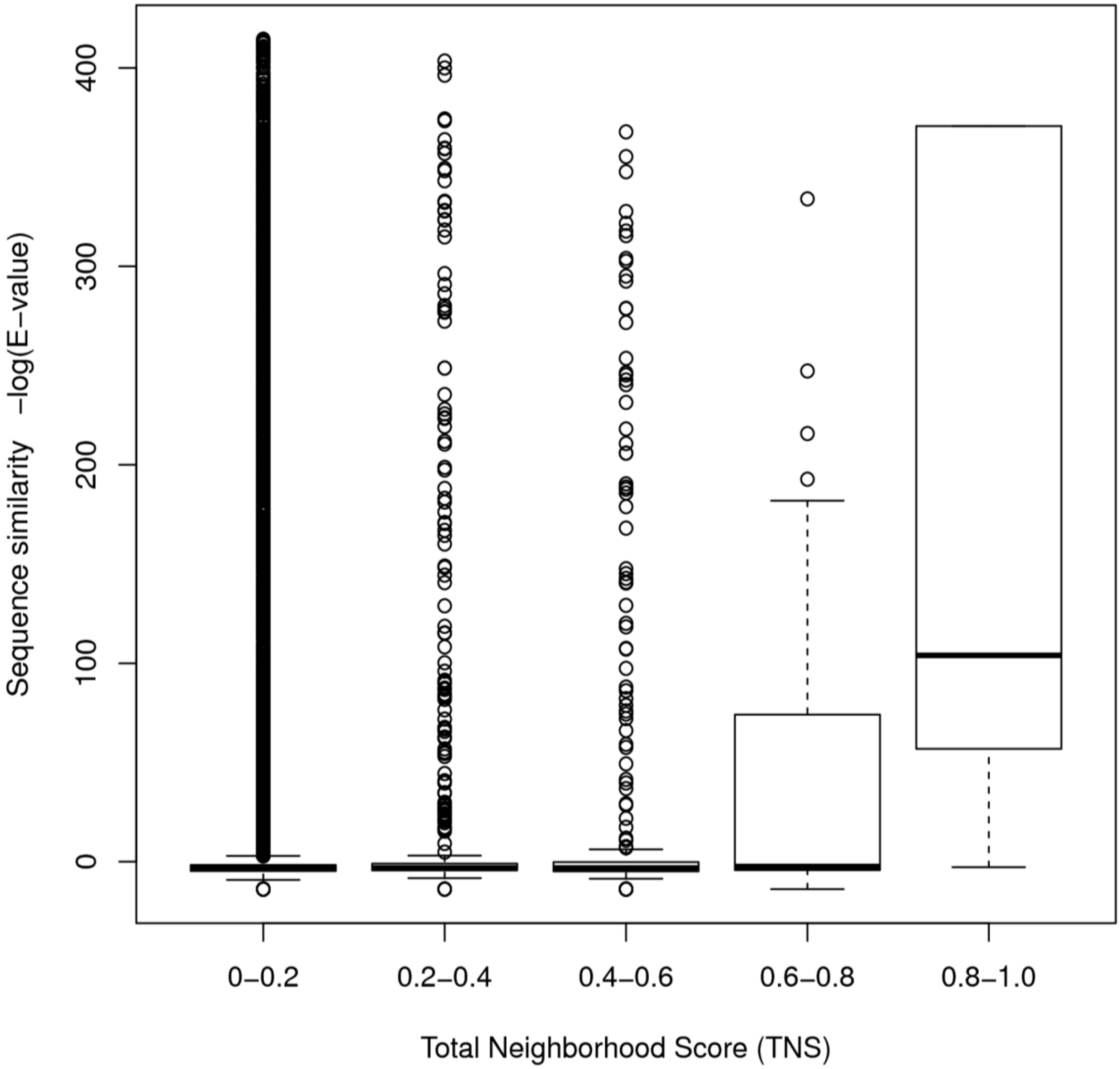

Figure S1: Box and whisker plots of the distribution of sequence similarity scores among genes within neighborhoods, stratified by the total neighborhood score computed from the Microarray Atlas data. Boxes show upper and lower quartiles and the dark line within the box is the median. The whiskers show the range of the data, while small circles denote outliers.

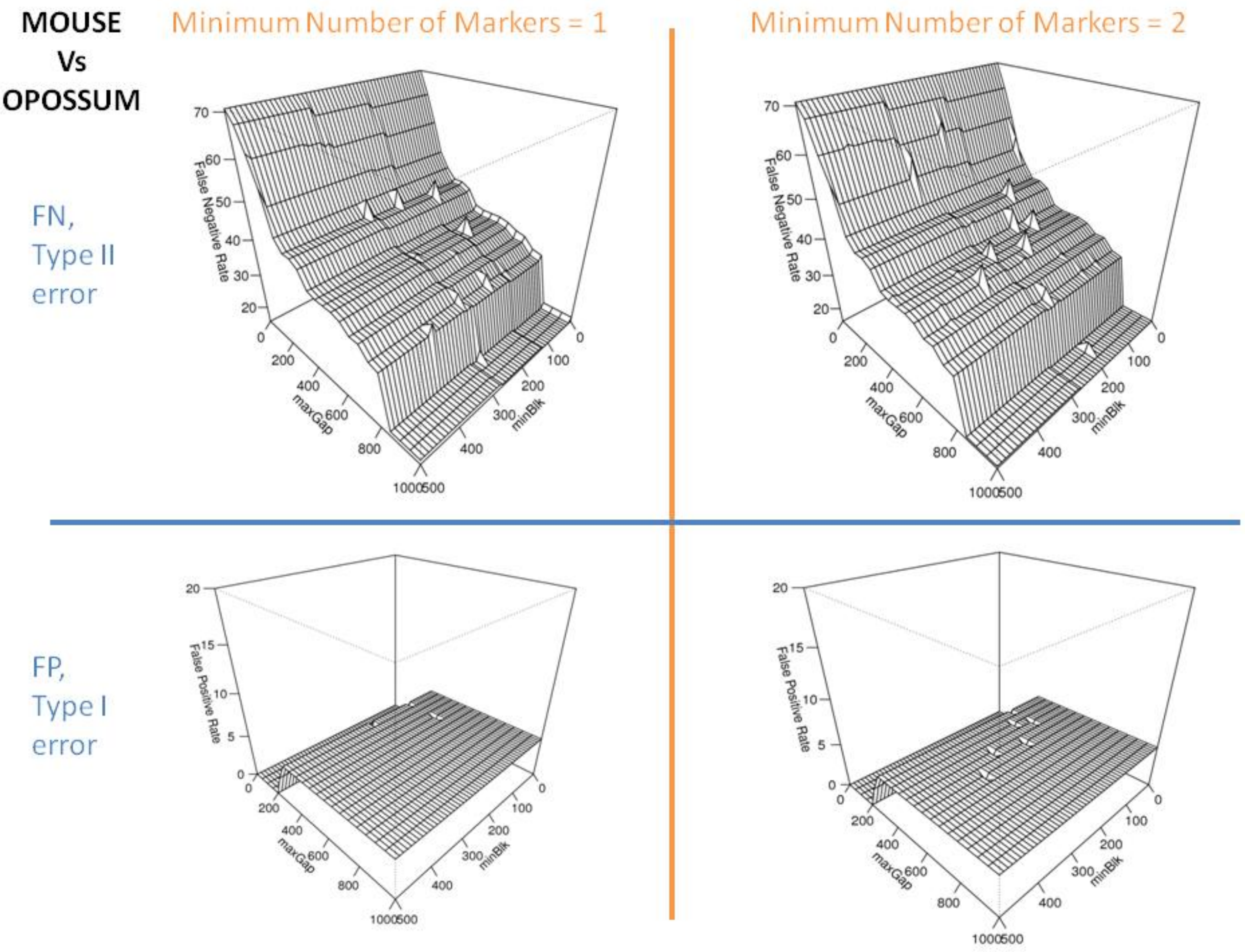

Figure S2. Effects of syntenic block parameter settings on error rates of gene neighborhood synteny detection between the Mouse and Opossum genomes

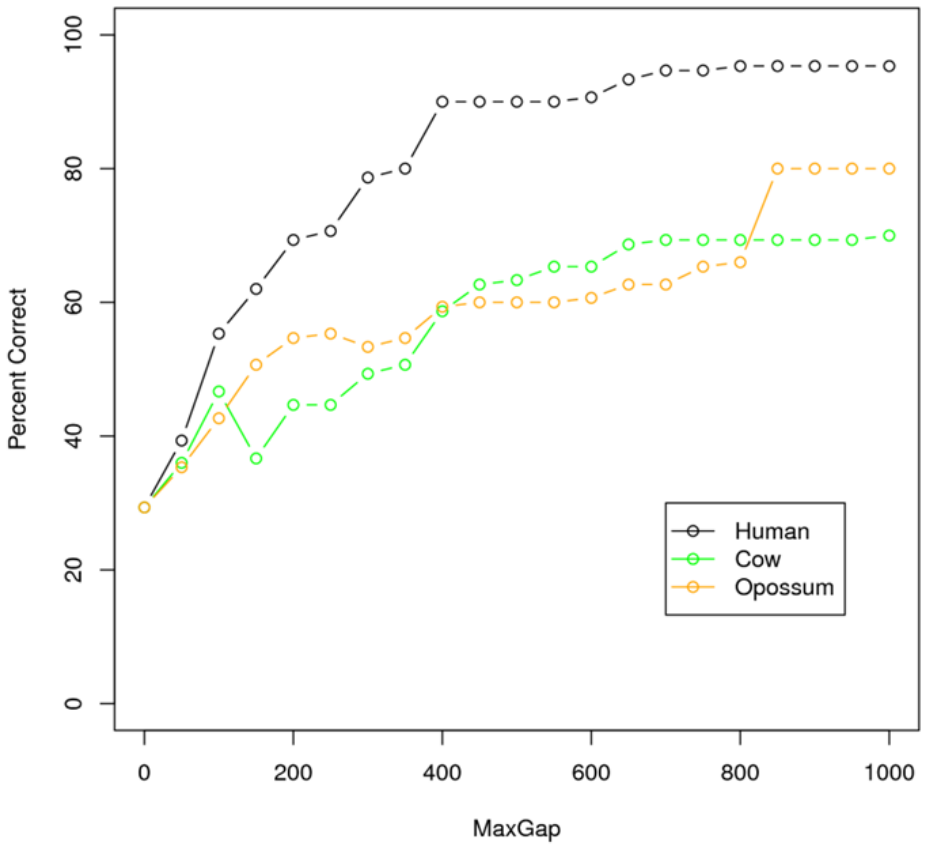

Figure S3. Effect of MaxGap parameter (in kb) on accuracy of gene neighborhood synteny

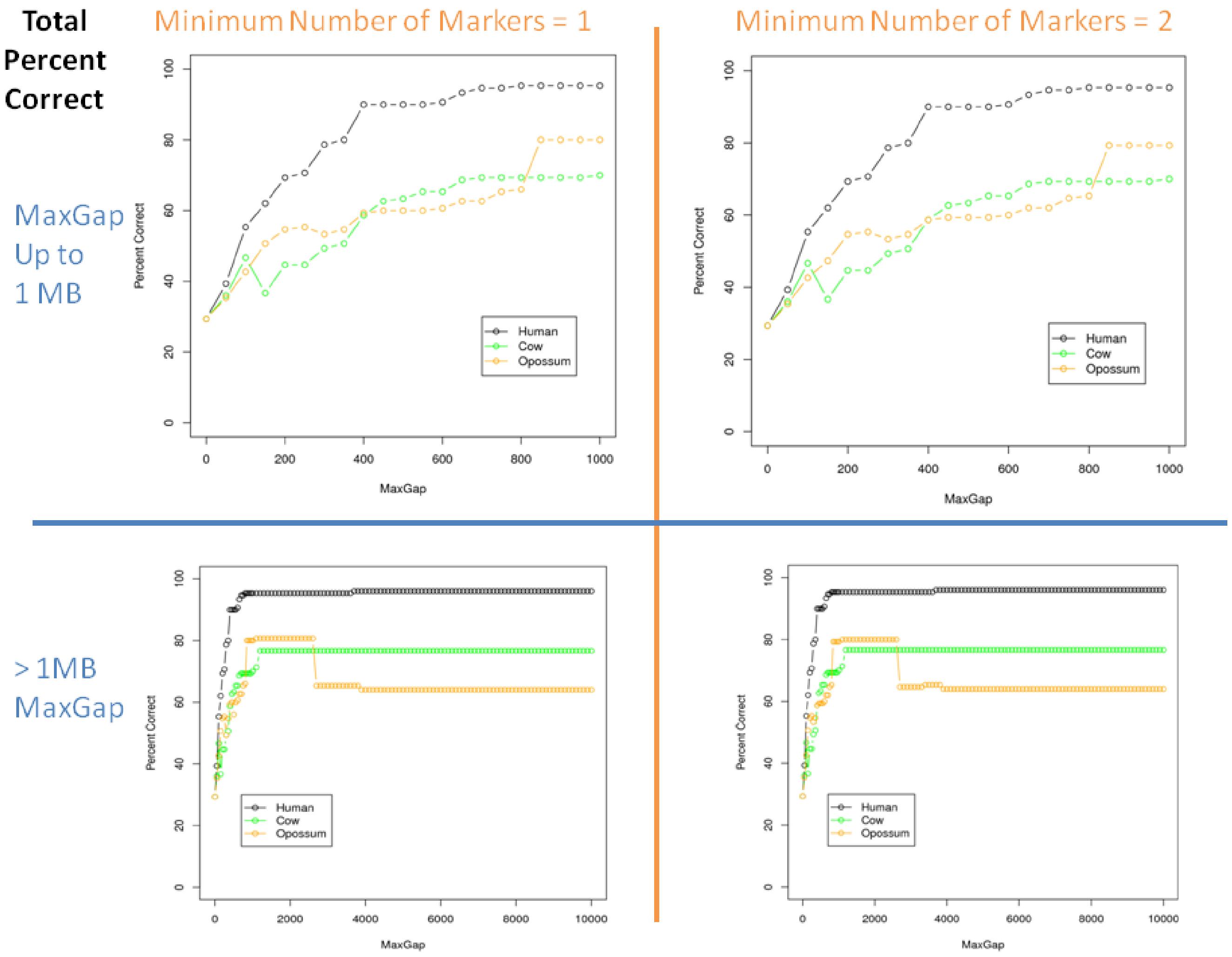

Figure S4. Effect of MaxGap parameter settings (in kb) on accuracy of gene neighborhood synteny
