# Supplementary material for: G-NEST: a gene neighborhood scoring tool to identify co-conserved, co-expressed genes
Source: BMC Bioinformatics. 2012 Sep 28;13:253. doi: 10.1186/1471-2105-13-253 (PMC3575404; doi:10.1186/1471-2105-13-253)
Supplement: Additional file 1 — G-NEST software. The Gene NEighborhood Scoring Tool (G-NEST) combines genomic location, gene expression, and evolutionary sequence conservation data to score putative gene neighborhoods across all window sizes. See README file for installation instructions. Note: the software requires PostgreSQL 9.0 or above. [file 1471-2105-13-253-S1.gz › gnest_params.doc]

G-NEST program usage

**gnest.pl** --option [value] …	[synteny_taxon_id synteny_file] …

for multiple option/value pairs (from the table below) and possibly multiple synteny_taxon_id/synteny_file pairs

| **Parameter** (*=required) | **Value type** | **Description** |
| --- | --- | --- |
| project_taxon_id * | integer | Taxon id of the species of analysis |
| project | Single token of lowercase letters, digits, and underscores | A name to be used for internal segmentation of the database (name for a schema) and as a prefix in the output file names. If omitted, a project name will be derived from the project_taxon_id and date. |
| chromosomes * | Single token alphanumeric | Chromosomes lengths file: chromsome, length. |
| genes * | file name | Genes info file (gene_name, chromosome, strand, start_pos, end_pos) |
| samples | file name | Samples info: sample_name, bio-state and replicate number. If omitted, each sample is treated as a separate bio-state with only one replicate |
| expr_data * | file name | Expression levels for gene_name X sample_name. |
| filter_on_mas5 | file name | MAS5 present/absent values for gene_name X sample_name, used to filter out absent genes from the analysis. If omitted, no such filtering is done. |
| filter_min_expr | floating point | Minimum expression threshold for a gene to be included in the analysis. If omitted, no such filtering is done. |
| min_win_size | integer | Size of smallest sliding window (in bp). Default=100000 |
| max_win_size | integer | Size of largest sliding window (in bp), must be a multiple of 'min_win_size'. Default=10000000 |
| min_gene_count | integer | Minimum number of genes in a neighborhood, for gene-count window analysis. |
| max_gene_count | integer | Maximum number of genes in a neighborhood, for gene-count window analysis. |
| num_permutations | integer | Number of iterations of shuffled genes. Default=1000 |
| dump_db | boolean (none) | Save a script to reproduce the database? |
| keep_project | boolean (none) | Override the default behavior to delete the project data from the database after generating output files. |
| no_synteny | boolean (none) | Suppress analysis related to syntenic regions. By default, analysis is done according to syntenic mappings pre-populated in the database or synteny files provided by the user. |
| reports_output, graphs_output, tracks_output | file name | Names of output files for the zipped reports, graphs, and tracks (respectively). This is intended for Galaxy use. If omitted, the names will be derived from the project name. |
